# Color stability and surface roughness of ormocer- versus methacrylate-based single shade composite in anterior restoration

**DOI:** 10.1186/s12903-022-02423-8

**Published:** 2022-09-27

**Authors:** Maha M. Ebaya, Ashraf I. Ali, Huda Abed El-Haliem, Salah Hasab Mahmoud

**Affiliations:** Operative Dentistry Department, Faculty of Dentistry, Algomhoria Street, Po (box) 35516, Mansoura, Aldakhlia Egypt

**Keywords:** Color, Surface roughness, Single-shade composite, Staining media

## Abstract

**Background:**

The blending ability of universal shade composites and their stability in the oral environment are of great concern in restoring anterior teeth. This study aims to evaluate and compare the color stability and surface roughness of two single-shade composite restorations, ormocer-based composite (OBC) and methacrylate resin-based composite (RBC), after storing them in different staining media.

**Materials and methods:**

In this study, two universal shade composite restorative materials were tested: a nanohybrid OBC (Admira fusion X-tra, Voco) and a supra-nanofilled RBC (Omnichroma, Toukyama). In total, 60 cylindrical centralized cavities (diameter: 5 mm, depth: 2 mm) were prepared in sound extracted-human central incisors and divided into two equal groups according to the restorative material used (n = 30). According to the storage media, the teeth of each group were divided into three subgroups (n = 10): artificial saliva, black tea, and cola. The restoration color was evaluated for all teeth at baseline and after four weeks of storage. The color stability (∆E) was measured using a reflective spectrophotometer (X-Rite, model RM200QC, Neu-Isenburg, Germany). The surface roughness (Ra) was evaluated using three-dimensional optical profilometry (Wyko, Model NT 1100, Veeco, Tucson, USA). Additionally, the extracted data were analyzed using two-way analysis of variance (ANOVA), one-way ANOVA and Student’s *t*-test.

**Results:**

In the baseline evaluation, there were no statistically significant differences with respect to color matching or surface roughness results between the two studied restorative materials. However, the differences were statistically significant after storing them in different media.

**Conclusion:**

Universal composites showed satisfactory color matching with different teeth colors and accepted surface smoothness, whereas the aging procedure exerted a negative effect on their color stability and surface characteristics.

## Introduction

Currently, resin-based composites (RBCs) are widely used for anterior teeth because of their satisfactory aesthetics, preservation of the tooth structure, low cost and good mechanical properties [1]. Technological progress in the development of RBCs has brought improvements in both filler and organic matrices. Changes in fillers, especially in the size, distribution, and type of particles, have occurred in the past few years and have enhanced the mechanical and optical properties of composites [2]. Moreover, monomer progression improves both the polymerization reactivity and the mechanical properties of the formed adhesive layer. Both Bis-GMA and UDMA dimethacrylates have become the primary monomers that are broadly utilized today for most dental composites. Because of its high viscosity, it is important to add low molecular weight monomers to accomplish suitable viscosity for the final formulation that is used clinically. These diluent monomers increase the water sorption, polymerization shrinkage, and discoloration of the composite resin. New monomers have been investigated, aiming to enhance the composite restorative material properties [3].

Ormocer is the acronym for organically modified ceramic. Their production is based on hydrolysis and polycondensation reactions (sol–gel processing) to create a molecule with a long-chain inorganic silica backbone and lateral organic chains [4]. The composites with ormocer are claimed to demonstrate a higher degree of conversion, minimal polymerization shrinkage, color stability, toughness, and increased surface hardness as a result of the formation of a more highly cross-linked polymer network. Another ormocer advantage would be the higher biocompatibility because the increased number of chemical bonds among the methacrylate groups would decrease the amount of free unreacted monomers in the polymer network [5].

Color matching of the composite restoration to the tooth structure and its stability has been a considerable challenge for a long time; hence, the RBCs were modified over the years, with enhanced optical properties and a higher availability of opaque/translucent shades [6]. The availability of different shades and technique sensitivities render shade selection a very complicated process, as it greatly depends upon the dentist’s skill, bias, and desired outcomes. Certain conditions elicit dentists to use trial and error, which can develop an unacceptable shade that needs the steps to be revamped at the expense of the dentist’s chair time. Hence, there is no standardization of the optical properties for these categories, and there may be unexpected or disappointing results [7].

These difficulties prompt researchers to return to simplifying and decreasing the number of shades based on color interactions. The chameleon or blending effect is the ability of dental materials to show color shifting toward the surrounding dental hard tissue color, which decreases the number of shade guide tabs and recompenses the color mismatches to some extent [8]. The simplification process of color matching starts with the group-shade composites until the production of a universal shade composite material that claimed to match different tooth shades*.*

Superficial staining of esthetic restorations has been reported as one of the main causes of failures leading to restoration replacement. Discoloration may occur by intrinsic factors related to the material and also by extrinsic factors, as absorption of pigments from food and drinks. Nevertheless even the most recent composite resin products, due to their resin matrix’s nature, still absorb more moisture than ceramics and are thus more prone to the penetration of various staining agents [9, 10]. In addition, one of the most critical factors that affect the aesthetics of the restoration is its surface topography, as a smooth surface enables better optical compatibility with the enamel tissue and surface gloss, along with the prevention of the staining and discoloration of the restoration [11].

Unfortunately, only scarce information is available on the color stability of universal shade composite materials based on their resin matrix’s nature differences, also their surface changes that happen after exposure to different beverages. Therefore, there is an increased need for further studies to understand their staining susceptibility and surface changes. Thus, the first null hypothesis was that the color acceptability of OBC and RBC restorations would not change after storage in staining solutions. The second null hypothesis was that the surface roughness of OBC and RBC would not be affected after storage in staining solutions.

## Materials and methods

Two single-shade composite restorative materials with their adhesives were used according to the manufacturer’s instructions. The full description of these materials is presented in Table 1.

**Table 1:** Materials evaluated in this study

Material	Type	Composition	Batch number	Manufacturer	Application procedure
Admira fusion X-tra	Nanohybrid ormocer based composite	Matrix: Resin ormocer Filler: Silicon oxide nano filler, glass ceramics filler (1 µm) Filler content: 84 (w/w)	1604218	VOCO GmbH, Cuxhaven, Germany	The restoration was applied at layers that are a maximum of 4 mm thick and adapted with a composite modeling instrument (CompoRoller,Kerr) and light-cured for 40 s
OMNICHROMA	Supra nano filled composite	11, 6- Bis-methacryl ethyl oxycarbonyl amino, UDMA, TEGDMA, Mequniol, Di-butyl hydroxyl toluene and UV absorber Filler content: 79 (w/w) of spherical silica-zirconia filler mean particle size: 0.3 µm	1602201	Tokuyama Dental, Tokyo, Japan	Resin composite was applied incrementally that are a maximum of 2.5 mm thick and adapted with a suitable instruments. each increment was photopolymerized individually for 20 s
Futurabond M+	Single bond universal LC	2-HEMA (10–25%), Bis-GMA) (10–25%),ethanol (10–25%), acidic adhesive monomer(10 MDP) 2.5–5%),UDMA(2.5–5%),catalyst, pyrogenic silicic acids pH 2.3	1929068	VOCO GmbH, Cuxhaven, Germany	One drop of the bond was put on a mixing palette, then the adhesive was applied evenly to the surfaces of the cavity and rubbed it in for 20 s with a disposable applicator, after that the adhesive layer was dried off with dry, oil-free air for at least 5 s in order to remove any solvents, then the adhesive layer was cured for 10 s
Palfique Universal Bond	Self-cured dental universal adhesive	Phosphoric acid monomer, Bis phenol A di (2-hydroxy propoxy) dimethacrylate), Bis-GMA, TEGDMA, 2-HEMA, (MTU-6). Silane coupling agent, peroxide, Borate catalyst, Acetone. Isopropanol and purified water	040EZ8	Tokuyama Dental, Tokyo, Japan	One drop of each bond bottle (A and B) was put on a mixing palette and mixed thoroughly with a disposable applicator, the application was completed within one minute since it had volatile solvents, after mixing the color had changed gradually, the application was completed within 3 min, then the adhesive was applied evenly to the surface then air dry for 30 s
Etchant Gel Vococid		35% orthophosphoric acid	7523	VOCO GmbH, Cuxhaven, Germany	
Etching Gel HV		39 wt% phosphoric acid	162E69	Tokuyama Dental, Tokyo, Japan	

## Methods

### Teeth selection

In total, 60 freshly extracted maxillary central incisors were chosen from the Oral Surgery Clinic, Faculty of Dentistry, Mansoura University for this study. The teeth were indicated for extraction for periodontal reasons and were extracted from a healthy patient after the signing (approval) of a written informed consent- form by the patient. All teeth were examined under a stereomicroscope (10× magnifications) to rule out the existence of fissures, fractures, carious lesions, restorations, and erosion or abrasive lesions. Thereafter, the teeth were scaled and polished using a rubber cup and pumice. The teeth were stored in distilled water and kept in a deep freezer for 24 h (− 10 °C) to avoid changes in the optical properties of the teeth [2].

### Cavity preparation

**Fig. 1:**
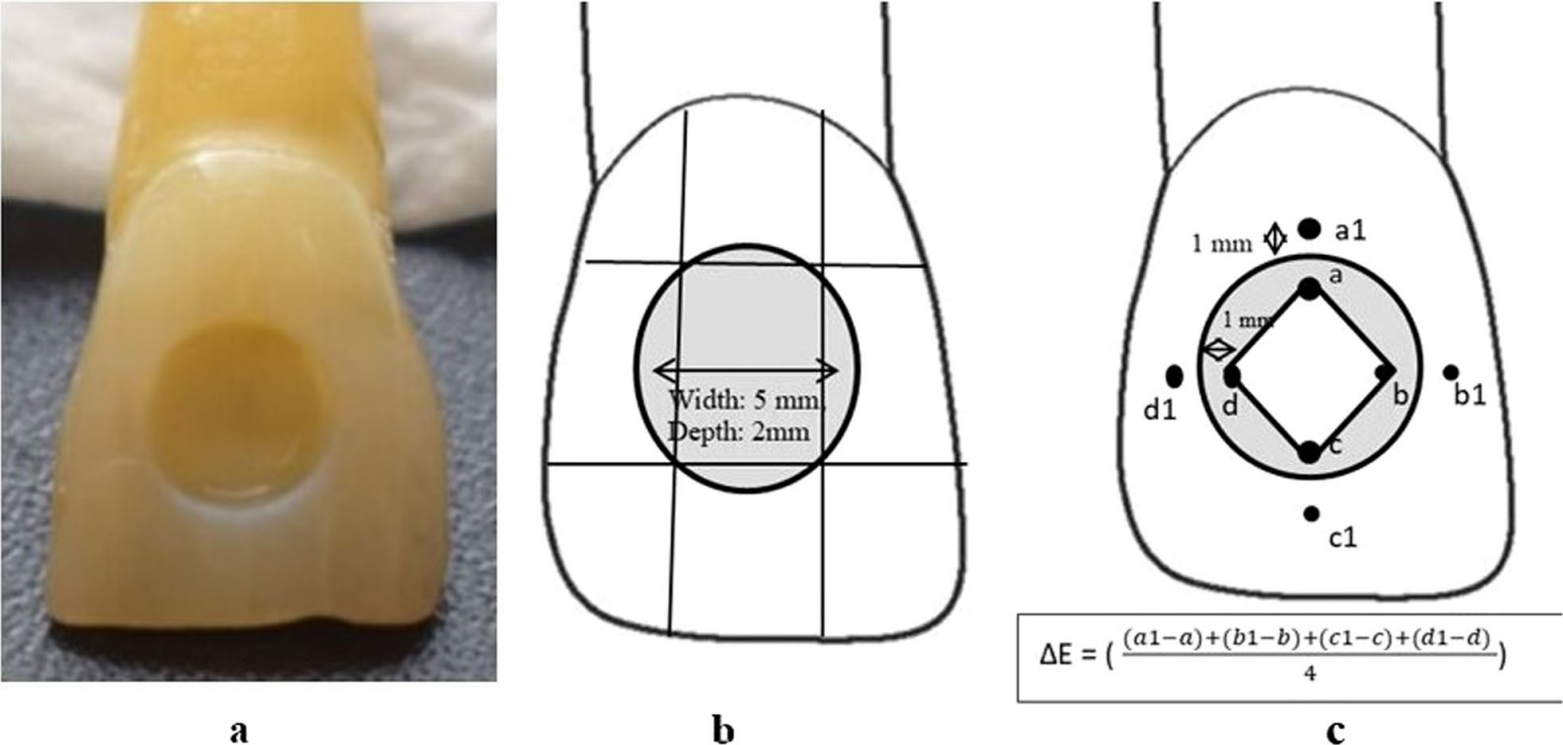
**a** Cavity preparation, **b** Cavity measurements, **c**: Color measurements; trapezoidal shape figure within the restoration with 4 apexes (**a**: cervical, **b**: mesial, **c**: incisal, **d**: distal) to represent all restoration colors, each one away 1 mm from restoration margin, while the other measuring points (**a1**, **b1**, **c1**, **d1**) located within tooth 1 mm away from the restoration margin.

Cylindrical shaped cavities (diameter: 5 mm; depth: 2 mm) were prepared at the center of the crown by dividing each tooth into three sections vertically and horizontally. In the middle part, a premeasured template was supported for a uniformly shaped outline for all the preparations (Fig. 1a, b) [12]. Cavity preparations were performed using carbide burs No. 330 on a high-speed handpiece with air/water coolant (W&H, SN 0,012,845); each bur was marked at 2 mm from its top and the final depth was checked by a periodontal probe. Importantly, each bur was used to preform five cavities. All cavosurface angles were kept at 90° without bevel designs. Two-thirds of the root was cut off, and the pulp chamber was blocked using resin composite.

### Specimen grouping

The prepared teeth were randomly divided into two equal groups (n = 30): group 1 was restored with nanohybrid OBC, and group 2 was restored with supra-nanofilled RBCs. All the restorative materials and their bonding systems were utilized according to the manufacturer's instructions. Additionally, they were finished immediately with a superfine diamond grinder (25 μm) attached to a high-speed handpiece at 200,000 rpm under water cooling systems for 10 s to simulate the initial tooth contour. Then, the teeth were polished with an aluminum oxide disc system (Sof-Lex, 3 M ESPE, 44-0007-7442-0-A lot N664515, St Paul, MN, USA). Numbers were assigned to each tooth to differentiate the teeth in each group during thermocycling and ensure the examination of the same tooth immediately and after aging procedures.

### Staining procedures

Each group was divided into three subgroups (n = 10), and each subgroup was further immersed in different storage media. The teeth were waterproofed with a colorless nail polish on their bucal and lingual surfaces. The staining solutions used were: artificial saliva, tea, and cola.

- *Subgroup (a)* The teeth were stored in 150 ml of artificial saliva (Sodium biphosphate 23%. Sodium chloride 11.8%, potassium chloride 11.8%, urea 29.5%, pH 6.9) that was prepared in the Pharmaceutics Department, Faculty of Pharmacy, Mansoura University.
- *Subgroup (b)* The teeth were stored in a black tea solution (Caffeine, tannins, theophylline, vitamin, glucose, pH 5.05, Black Tea Lipton, UK) that was prepared by immersing 1 prefabricated tea bag into 100 ml of boiling distilled water for 5 min.
- *Subgroup (c)* The teeth were stored in cola soft drink (Carbonated water, high fructose corn syrup, caramel color, coca flavor, phosphoric acid, caffeine, pH 2.5, Coca-Cola Co., USA). The containers’ lids were closed tightly to prevent the leaking of carbonic gas to maintain an acceptable carbonic gas level, and a new bottle was used daily.

The specimens were kept in staining solutions and further in an incubator (BTC, Model: BT1020, Egypt) at 37 °C over a 28-days test period [13]. Additionally, a digital pH- meter (CONSORT nv, Parklaan 36, B2300 Turnhout, Belgium) was used to measure the pH of fresh immersion liquids. Each specimen was rinsed for 30 s in distilled water and cleansed gently with a soft bristle toothbrush to expel any loose sediment caused by the immersion solution during the incubation period. The same rinsing process was repeated daily. Furthermore, the three storage liquids were refreshed daily to prevent microbial growth and kept in vials with lids that inhibited the evaporation of staining solutions. All the teeth were handled carefully from the root to prevent any surface scratches.

The specimens were thermocycled (SD Mechatroniks thermocycler, Germany) for 3500 cycles between water paths held at 5 °C and 55 °C with 15 s dwell time in each bath [14]. Thereafter, the teeth were rinsed gently with distilled water and air-dried.

### Color stability evaluation

For each group, the baseline specimen color was measured by a reflective spectrophotometer (X-Rite, model RM200QC, Neu-Isenburg, Germany). Color measurements were performed under 45°/0° geometry, the spectrophotometer image capture was affined to D65 Standard illumination and 2° Standard Observer. The teeth were aligned with the device and the aperture size was adjusted to 4 mm. The measurements were conducted using the Commission Internationale de I'Eclairage (CIE) L (lightness), C (chroma) and H (hue) over a white background. The color change values of the specimens were estimated according to the CIEDE2000 formula:

$$\Delta {\text{E}}_{00} = \sqrt {\left( {\frac{{\Delta {\text{L}}\prime }}{{{\text{K}}_{{\text{L}}} {\text{S}}_{{\text{L}}} }}} \right)^{2} + \left( {\frac{{\Delta {\text{C}}\prime }}{{{\text{K}}_{{\text{C}}} {\text{S}}_{{\text{C}}} }}} \right)^{2} + \left( {\frac{{\Delta {\text{H}}\prime }}{{{\text{K}}_{{\text{H}}} {\text{S}}_{{\text{H}}} }}} \right)^{2} + {\text{R}}_{{\text{T}}} \left( {\frac{{\Delta {\text{C}}\prime }}{{{\text{K}}_{{\text{C}}} {\text{S}}_{{\text{C}}} }}} \right)\left( {\frac{{\Delta {\text{H}}\prime }}{{{\text{k}}_{{\text{F}}} {\text{S}}_{{\text{H}}} }}} \right)} ,$$

where ΔE_00_ is the color difference, ΔL, ΔC, and ΔH are the differences in lightness, chroma, and hue, respectively, for a pair of specimens in CIEDE2000, and R_T_ is the rotation factor that considers the interactions among hue and chroma differences in the blue area. Weighting functions, viz. S_L_, S_C_, and S_H_, modulated the total color difference for variation in the site of the color difference pair in the L*, a*, and b* coordinates, and the parametric factors, K_L_, K_C_, and K_H_ were the expressions for the experimental conditions [15]. The CIEDE2000 parametric factors of the color difference formula were adjusted to 1. Furthermore, the perceptibility threshold was adjusted at ΔE_00_ ≤ 0.8 units, and the clinical acceptability threshold was adjusted at ΔE_00_ ≤ 1.8 units [16].

The color was measured at four fixed points (diamond shape) on the restoration and their adjacent points on the tooth surface, and an average of these readings was taken to represent each restoration color (Fig. 1c). After storage and thermocycling, the specimen color was examined in the same manner. Next, ΔE between the delayed and baseline results was established.

### Surface roughness evaluation

A 3D optical profilometer noncontact (Wyko, Model NT 1100, Veeco, Tucson, USA) attached to a PC with image software (Vision 32, Veeco, USA) was used to measure the surface roughness [17]. The software used for creating the images supplied arithmetic roughness mean (Ra) data based on the peaks and valleys exhibited in the analyzed area using profilometer with 0.8 mm cut off and 2.4 mm evaluation length. Thus, a 3D image of the specimen surface profile was produced. Thereafter, five 3D images were gathered for each specimen in the central and side areas of 10 µm × 10 µm.

Data were tabulated, coded, and anatomized with the Statistical Package for Social Science (SPSS) computer program version 26.0 to produce the descriptive data. The calculation of descriptive statistics was conducted in the form of mean and standard deviation (SD). Two-way analysis of variance (ANOVA) followed by Bonferroni post- hoc test was used to detect the effect of restorative materials and coloring media on color stability. One-way ANOVA followed by post- hoc Tukey’s test was used to define the statistically significant differences between the restorative materials kept in each medium. Additionally, Student’s *t*-test (paired, unpaired) was utilized to compare the mean values of parametric data between the two groups (P value < 0.05 was considered statistically significant).

## Results

### Color stability results

Two-way ANOVA test outcomes exhibited no significant interaction between the restorative material type and the coloring agents used (P = 0.536). One-way ANOVA for OBCs and RBCs was established statistically significant differences between the specimens after storage in different coloring media (P < 0.001) and (P < 0.0016) respectively. Afterwards, the Tukey test illustrated these differences among subgroups, as there was a significant difference between cola and saliva (P < 0.01); however, there were differences among tea and saliva subgroups, and tea and cola subgroups which were not significant (P ≥ 0.05). In OBC (mean ∆E00 for saliva = 6.21 ± 1.85 < for tea = 7.27 ± 2.73 < for cola = 9.13 ± 1.37) which were significantly higher than those for RBC, the mean ∆E_00_ for saliva = 4.43 ± 2.11 < for tea = 6.34 ± 1.60 < for cola = 6.83 ± 1.71). These ∆E_00_ values are summarized in Table 2.

**Table 2:** Mean and standard deviation for the color stability of the two studied universal shade composites

	Ormocer-based composite		Methacrylate-based composite	
	ΔE-baseline	ΔE-delayed	ΔE-baseline	ΔE-delayed
Saliva	2.22 ± .63	6.21 ± 1.85^#^	1.80 ± .52	4.43 ± 2.11^#^
Tea	2.14 ± .49	7.27 ± 2.73^#^	2.28 ± 1.05	6.34 ± 1.60^#^
Cola	2.42 ± .75	9.13 ± 1.37^#a^	2.28 ± .68	6.83 ± 1.71*^#a^

Data are expressed as the mean ± SD

*SD* standard deviation; *P* Probability significance when < 0.05

^a^Significance between Saliva & Cola

*Significance between ΔE**-**ormocer-based composite vs ΔE-methacrylate-based composite either at baseline or delayed time (Test used: unpaired student’s *t*-test)

^#^Significance between ΔE-baseline versus ΔE-delayed either ormocer-based composite or methacrylate-based composite (Test used: paired student’s *t*-test)

### Surface roughness evaluation results

**Fig. 2:**
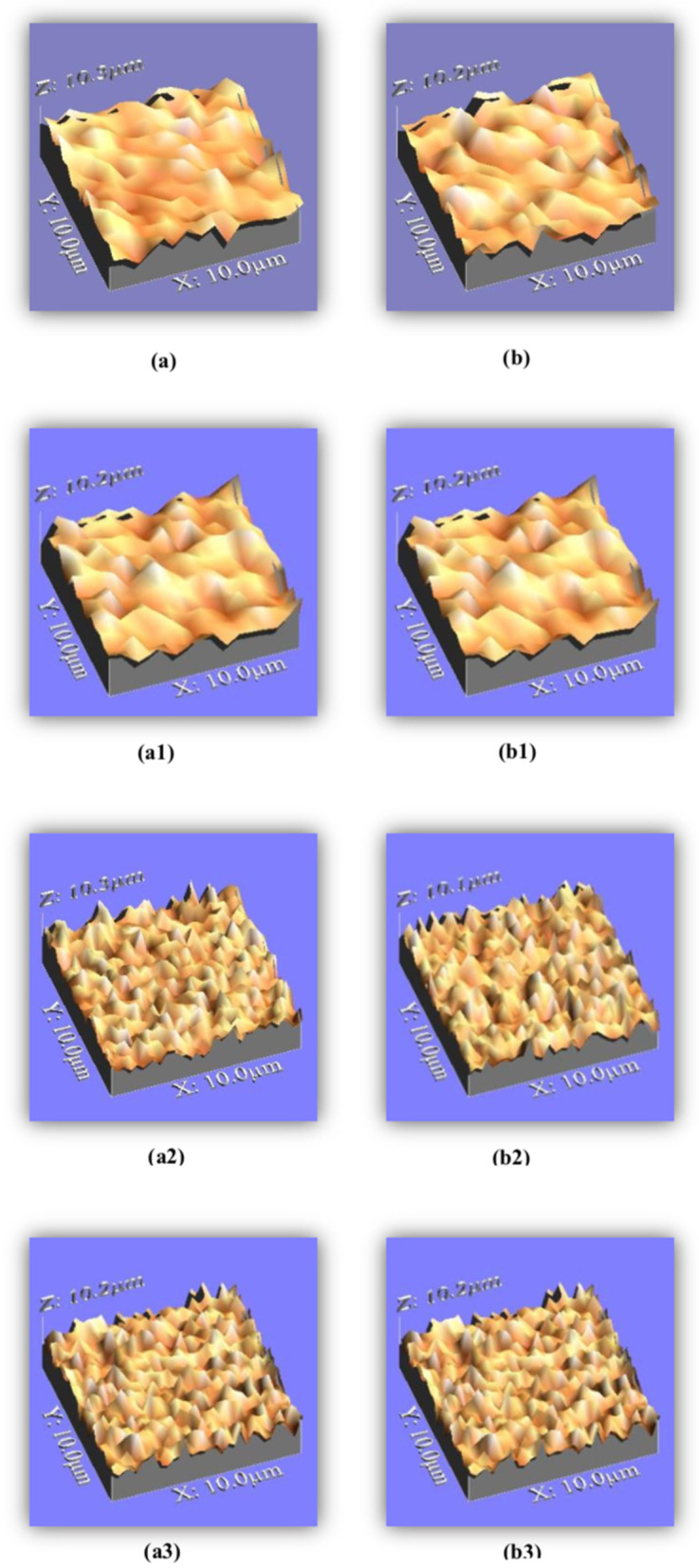
Surface topography of universal shade composites. **a** OBC immediately, a1, OBC after storage in saliva, a2. In tea, a3. In cola, **b** RBC immediately, b1. In saliva, b2. In tea, b3. In cola

Two-way ANOVA results showed no significant interaction between the tested composite materials and coloring media (P = 0.75). One-way ANOVA for OBC established statistically significant differences among specimens in different coloring media after storage (P < 0.048). Afterwards, the Tukey test showed the differences among the subgroups, as there were significant differences between cola and saliva (P < 0.045), whereas no significant differences were established between the other subgroups (P > 0.05). For RBC, statistically significant differences were established after storage in coloring media (P < 0.001), and the Tukey test showed statistically significant differences between the subgroups cola and saliva, along with tea and cola (P < 0.05), whereas no statistically significant difference manifested between the subgroup of tea and saliva (P > 0.52). Student’s paired *t*-test showed significant differences between the baseline and delayed results for each restoration (P < 0.05), except for the saliva subgroup, whereas Student’s unpaired *t*-test showed no significant differences between the baseline results of materials or their delayed results (P > 0.05). These outcomes are summarized in Table 3 and Fig. 2.

**Table 3:** Mean and standard deviation for the surface roughness of the two studied universal shade composites

	Ormocer-based composite		Methacrylate-based composite	
	Ra-baseline	Ra-delayed	Ra-baseline	Ra-delayed
Saliva	0.253 ± 0.0023	0.253 ± 0.0046	0.254 ± 0.0031	0.255 ± 0.0009
Tea	0.252 ± 0.0035	0.254 ± 0.002^#^	0.255 ± 0.002	0.256 ± 0.0014
Cola	0.253 ± 0.003	0.257 ± 0.002^#b^	0.256 ± 0.0019	0.258 ± 0.0017^#bc^

Data are expressed as the mean ± SD

*SD* standard deviation; *P* Probability significance when < 0.05

^a^Significance between Saliva & Tea

^b^Significance between Saliva & Cola

^c^Significance between Tea & Cola (Test used: one way ANOVA followed by post-hoc Tukey)

*Significance between Ra-baseline or Ra-delayed either for ormocer-based composite versus methacrylate-based composite either at baseline or delayed time. (Test used: unpaired student’s *t*-test)

^#^Significance between Ra-baseline and Ra-delayed either for ormocer-based composite or methacrylate-based composite (Test used: paired student’s *t*-test)

## Discussion

Creating an aesthetic anterior composite restoration has been an exacting challenge for a long time because of the limitations of many materials that affect either shade integration or surface quality and probably color stability. In addition to the drawbacks in technology, a lack of predictability and certain complexity in the clinical application were inherent to the technique and produced its elitist for a long time. Universal shade composites claimed to have a breakthrough in dentistry that has an impact on treating all these problems [18].

Universal shade composites are based mainly on structural color phenomena. Structure colors are the result of the fundamental optical processes of interference, scattering or diffraction. Subsequently, unlike traditional pigmented color that comes from the light absorption of pigments, structural color claimed to be more adequate and stable. In this study, two omni-chromatic universal shade composites were chosen with different organic matrix structures, as the matching between refractive indexes between the organic matrix and the fillers is one of the fundamental issues to achieve structural color [19]. Moreover, the OBC used some pigments in its structure, while RBC color was purely based on the structure color concept.

In this study, the employed methodology was in accordance with previous studies. Every effort made to ensure the standardization of the methodology, as well as all the steps, was performed by a single operator. The extracted human teeth were used as the studied materials to gain their color by induction from the surroundings. In addition, natural teeth have different optical properties, so they were used to reveal the clinical conditions. The series of Sof-lex polishing discs was the system of choice. Aluminum oxide discs have been suggested as a standard protocol because of their capability to form smooth, nondestructive polished surfaces that are less susceptible to chemical solubility [20]. The studied liquids were chosen as colorant agents because of their constant consumption in daily life. A four-week immersion period was chosen, which is equal to approximately 2.5 years of clinical aging (24 h of staining in vitro corresponds to about 1 month in vivo, and 3500 thermal cycles were performed to mimic the oral environment during this period) [14].

Spectrophotometry and the CIEDE2000 (Δ*E*_00_) are recommended methods for dental purposes. Several studies suggested that the Δ*E*_00_ observed that it is used provided higher degree of fit than the ΔE*ab improving the correlation between visual color differences and calculated differences. As it includes not only lightness, chroma, and hue weighting functions, but also an interaction term between chroma and hue differences [15]. The 50:50% acceptability threshold (AT) is used in dentistry to compare calculated CIE L*, a*, b* color differences to actual visual color analysis. The AT is the point at which 50% of visual examiners would consider an object as an acceptable match to another object. In this study, AT was used to interpret results and to determine clinical significance [21].

A noncontact digital profilometer microscope was used because of its ability to scan the surface with a type of laser and provide a 3D surface map without damaging the specimens, thereby proving to be a fast and easy evaluation method [22]. The surface roughness over the roughness threshold (Ra = 0.2 µm) causes a simultaneous increase in biofilm accumulation, and no further decrease in bacterial adhesion could be observed under the threshold value [17]. Smooth surfaces add to the comfort of the patient as a surface roughness change of 0.3 µm can be identified by the tip of the tongue [23].

Based on this study’s results, the color differences for OBC and RBC are considered clinically acceptable color match. This finding may be attributed to the unique pure silicate technology of OBC restoration, as manufacturers claimed that its nanoparticulate amplifies the chameleon effect, further reinforcing its ability to blend and adapt to the surrounding tooth structure because it is smaller than the wavelengths of visible light. Therefore, its nanoparticulate neither diffracts nor refracts light, but allows the light to pass through uninterrupted and bounce off the surrounding tooth structure.

Moreover, the smart chromatic technology of RBCs, a unique technology based on fillers (uniform supra-nano spherical fillers and round fillers, fabricated with zirconium dioxide and silicon dioxide) that are claimed to generate red to yellow structural color, as light passes through the fillers, reflects the red to yellow range of colors found in all the teeth. These colors then combine with the surrounding tooth color, thus permitting the unprecedented ability of color matching. Consequently, the cured composite blends with the surrounding tooth structure.

These results agreed with those of Bakti et al. [24] who concluded that nanofilled RBCs exhibit a chameleon effect whereby they can adjust their color to suit that of their surroundings. They also concluded that the chameleon effect has a limitation in its color adjustment. Additionally, Abdelrouf et al. [25] assessed the visual color matching and blending effect of universal RBCs and concluded that universal composites showed an acceptable color matching, but it may not be the optimal selection when esthetic is the patient’s prime concern. These results may occur due to the differences in the materials used. In contrast, de Abreu et al. [26] and Iyer et al. [27] reported that the color matching of single-shade composite is inferior to that of multishade composite, which may limit their clinical use in the cases of high esthetic demand.

After the staining process, the first null hypothesis was rejected as immersion in staining media had showed a diverse effect on color of the two tested materials, which was clinically unacceptable. Color changes can be attributed to the combination of matrix degradation by acids, penetration/absorption of colorants into the material as well as the surface adhesion/adsorption of colorant [28]. Immersion in saliva may lead to a yellowish color for restoration because it contains mucin [9]. Additionally tea is rich in tannins, which promotes yellowish staining as it enhances the chromogens’ ability to adhere to the materials’ surfaces; moreover, immersion in tea increases the surface roughness, hence causing further stain updates [29]. Studies have shown that black tea and tannin-containing compounds cause chemical reactions due to the presence of denaturing factors that lead to stable discoloration [30]. Cola subgroups had the highest color change which promoted no longer acceptable color changes. As cola is a yellow–brown carbonated beverage, staining is caused due to sulfite ammonia caramel. It also has a decolorizing effect that affects the sorption and solubility of RBC material [31, 32].

Additionally, the effect of thermocycling, is a combination of thermal and hydrolytic degradation, and is considered a method that simulates temperature-related breakdown by sudden repeated changes in temperature, thereby affecting the durability of the material. Water absorption impacts the mechanical characteristics of composites toward hydrolytic degradation. It can also lead to microfractures in the interface between the resin matrix and the fillers and induce superficial stress due to high temperature gradient differences, which are close to the surface and affect its roughness and the ability to gain stains [33].

These results were in accordance with those of Reddy et al. [34] and Ozkanoglu et al. [35] who reported that in vitro staining affects the color match of esthetic restorations. They also concluded that the staining intensity of cola is greater than that of tea. Additionally, Pordan et al. [36] assumed that specimens immersed in saliva exhibited color changes compared to baseline, and these changes were attributed to the water sorption characteristics of the restorations. In contrast, the results of previous studies were not in agreement with this study [30, 31], that is; tea had a higher staining ability than that of cola.

With regard to surface roughness evaluation, OBC and RBC were considered clinically acceptable in terms of bacterial adhesion and patient comfort. These results may be attributed to the fact that the manufacturing of the nanoparticles in both materials was the same which is called the sol–gel process. This process is a controlled reaction between different chemistries that results in the creation and growth of uniform nanospheres (nanoparticles) that are harvested once they grow to the desired diametrical size, (the nanoparticles’ diameter in OBC = 20–40 nm), whereas RBC fillers were based on their own patented “Sub-Micro-Pearl-Technology”. In this process, the sol–gel method is used to progressively coat the spherical fillers in an organic solution. After several weeks, the fillers have “grown” evenly in a spherical shape and are exactly 0.26 µm in size. This feature results in a highly smooth polished surface [15].

This result was in accordance with that of Cunha et al. [37] and Gurbuz et al. [38] who concluded that OBC did not present significant differences compared to the surface roughness of the conventional composites because of the comparable filler size and load between them. However, Tagtekin et al. [39] concluded that ormocer had a higher surface roughness than conventional hybrid RBC as the filler particles in the used ormocer are harder than the matrix, causing preferential loss through finishing and polishing, as well as leaving the filler phase in a positive surface and causing more surface roughness.

After staining, the second null hypothesis was rejected as differences in surface roughness after storage were occurred. These results were in agreement with previous studies [40] and may be ascribed to the chemical erosion from tea as it contains oxalic, malic, and citric acid with a pH (value of 5.4), which is acidic in nature. Additionally, cola has a low pH (value of 2.5) that influences the surface integrity of the RBC, thereby leading to an increasing surface roughness. The lower pH of cola with respect to tea can explain the significant difference between their RBC results [34, 41].

The present study had some limitations. One of these was the lack of information to verify the capacity of these resin-based composites on mimicking different surroundings. Furthermore, the effect of tooth shades on their blending capacity. In addition, all clinical conditions are difficult to replicate with high precision in a laboratory study; subsequently, further clinical investigations are still necessary to predict the acceptability and longevity of universal shade composite restorations.

## Conclusion

Within the limitations of this in vitro study, the following conclusions can be drawn:

1. Universal shade composites have a satisfactory color matching to different teeth colors and accepted surface roughness immediately.
2. Both ormocer- and methacrylate-based composites are prone to unacceptable color changes and surface roughness after aging.
3. Cola soft drink had the highest adverse effect on the restorations color and surface roughness.

## Data Availability

The datasets used and/or analysed during the current study available from the corresponding author on reasonable request.
